# Observation of behavioural skills by medical simulation facilitators: a cross-sectional analysis of self-reported importance, difficulties, observation strategies and expertise development

**DOI:** 10.1186/s41077-023-00268-x

**Published:** 2023-11-29

**Authors:** Lars Mommers, Daniëlle Verstegen, Diana Dolmans, Walther N. K. A. van Mook

**Affiliations:** 1 Department of Simulation in Healthcare, Maastricht University Medical Centre, PO 5800, NL-6202 AZ Maastricht, The Netherlands; 2 Department of Anaesthesiology and Pain Medicine, Maastricht University Medical Centre, Maastricht, The Netherlands; 3 School of Health Professions Education, Maastricht University, Maastricht, The Netherlands; 4 Department of Postgraduate Medical Training, Maastricht University Medical Centre, Maastricht, The Netherlands; 5 Department of Intensive Care Medicine, Maastricht University Medical Centre, Maastricht, The Netherlands

**Keywords:** Behavioural skills, Soft skills, Nontechnical skills, Current practice, Self-reported competence, Simulation, Interprofessional education, Team training

## Abstract

**Background:**

The association between team performance and patient care was an immense boost for team-based education in health care. Behavioural skills are an important focus in these sessions, often provided via a mannikin-based immersive simulation experience in a (near) authentic setting. Observation of these skills by the facilitator(s) is paramount for facilitated feedback with the team. Despite the acknowledgement that trained facilitators are important for optimal learning, insight into this observation process by facilitators is limited.

**Objectives:**

What are the self-reported current practices and difficulties regarding the observation of behavioural skills amongst facilitators during team training and how have they been trained to observe behavioural skills?

**Methods:**

This cross-sectional study used a pilot-tested, content-validated, multi-linguistic online survey within Europe, distributed through a non-discriminative snowball sampling method. Inclusion was limited to facilitators observing behavioural skills within a medical team setting.

**Results:**

A total of 175 persons filled in the questionnaire. All aspects of behavioural skill were perceived as very important to observe. The self-perceived difficulty of the behavioural skill aspects ranged from slightly to moderately difficult. Qualitative analysis revealed three major themes elaborating on this perceived difficulty: (1) not everything can be observed, (2) not everything is observed and (3) interpretation of observed behavioural skills is difficult. Additionally, the number of team members health care facilitators have to observe, outnumbers their self-reported maximum. Strategies and tools used to facilitate their observation were a blank notepad, co-observers and predefined learning goals. The majority of facilitators acquired observational skills through self-study and personal experience and/or observing peers. Co-observation with either peers or experts was regarded as most learn some for their expertise development. Overall, participants perceived themselves as moderately competent in the observation of behavioural skills during team training.

**Conclusions:**

Observation of behavioural skills by facilitators in health care remains a complex and challenging task. Facilitators’ limitations with respect to attention, focus and (in)ability to perform concomitant tasks, need to be acknowledged. Although strategies and tools can help to facilitate the observation process, they all have their limitations and are used in different ways.

**Supplementary Information:**

The online version contains supplementary material available at 10.1186/s41077-023-00268-x.

## Background

The global interest in so-called behavioural skills increased significantly over the past decades. This was especially due to the association between behavioural skills and preventable medical errors, the latter resulting in unnecessary patient morbidity and mortality [1–5].

Behavioural skills or non-technical skills (NTS) can be defined as the cognitive, social and personal skills that complement ‘technical skills’ and contribute to safe and efficient task performance [6]. C*ognitive* NTS is often subdivided into situation awareness, decision-making, coping with stress and managing fatigue, whereas *social* NTS consists of task management, leadership, communication and teamwork [7, 8]. Related terms for NTS commonly found in literature are ‘crew’ or ‘crisis resource management’ (CRM), risk management, ‘soft skills’ and ‘crisis avoidance resource management’. Murphy et al. [9] proposed the use of ‘behavioural skills’ as a new lexicon over the term NTS as more accurately describing the range and complexity of interpersonal skills.

With the acknowledgement of behavioural skills’ importance in the prevention of medical errors, many team training programs focussing on these aspects were developed. Currently, well-established literature is available which underscores the importance of team-based behavioural skills training [10, 11] and evidence correlates these interventions to improved team performance and even improved patient outcomes and reduced mortality [12–17].

Commonly, these team-based trainings are offered through ‘immersive simulation’, during which teams are confronted with a scenario-based mannikin in a setting as authentic as possible. Feedback is usually facilitated in the debriefing phase by the facilitator. This debriefing phase is paramount in simulation-based training as this is where most learning occurs [18–20]. Savoldelli et al. [21] found that simulation training *without* feedback did *not* lead to improvement of behavioural skills in anaesthesia trainees. Furthermore, a meta-analysis showed properly facilitated debriefings to be three times more effective compared to non-facilitated debriefings [22]. Additionally, self-assessment of behavioural skills by participants is reported to be difficult, underscoring the importance of trained facilitators to guide this paramount process of debriefing [22–24].

Roussin and Weinstock [25] described a classification for the broad range of simulation-based training programmes currently available. The so-called zone 3 and 4 training entities encompasses ‘native team’ training (i.e. without role-playing), with a focus on behavioural skills and a facilitator (rather than an instructor) to guide the (post-event) debriefing. This is the type of training meant wherever this article refers to as ‘team training’.

### Problem definition

The process of ‘facilitated feedback’ during debriefing encompasses different stages: first of all, facilitators have to make observations of the behavioural skills. Then, they have to interpret and evaluate these observed aspects before they can facilitate a feedback conversation with the team. Despite the acknowledgement that trained faculty is paramount for optimal learning, insight into *how* facilitators observe behavioural skills and how their training can impact behavioural skill observation, and therefore, participants’ learning is still limited [26]. The available literature has mainly focused on ‘standardising’ this observation process at the end, by providing facilitators with tools, e.g. ‘behaviourally anchored rating scales’, to assess team performance [27]. This however does not describe *what* facilitators find important to observe, nor their reasoning and interpretation. More so, observation and provision of feedback in behavioural skills can be regarded as deceptively ‘simple’ due to the availability of many easy-to-use frameworks [8]. The contrary is the case, as feedback on behavioural skills remains challenging and requires trained faculty, even when applying robust frameworks [10, 28–31]. The process of becoming trained and experienced faculty in observing behavioural skills can be envisaged to span many years. However, such ‘expertise development’ studies regarding the process of behavioural skills feedback (i.e. observation, interpretation, evaluation and feedback) are scarce [32].

To summarise, experienced faculty is essential for the observation of behavioural skills and feedback and therefore team learning. However, sufficient knowledge of contemporary practice on the observation of behavioural skills by simulation facilitators, as well as potential areas for improvement is still limited.

### Objectives

This study aims to answer the following overall research question:

- ‘What are the self-reported current practices and difficulties regarding observation of NTS amongst facilitators during team training and how have they been trained to observe these skills?’

The study addresses the following three sub-questions:

1. *Which aspects of NTS find health care facilitators most important and/or most difficult to observe and why?*
2. *What strategies and tools (e.g. checklist) do health care facilitators utilise in their daily practice?*
3. *How are health care facilitators trained for observing NTS and what kind of future training needs do they have?*

## Methods

### Study design

This study used a cross-sectional methodology using a multi-linguistic online survey within Europe. The survey and all corresponding documents (i.e. participants’ information) were made available in four languages (Dutch, English, German and French).

### Participants

Facilitators supervising NTS team training sessions—previously mentioned SimZones 3 or 4—in healthcare were allowed to enrol in the study. Since the SimZones classification model might not be widely known, inclusion criteria were described as being a ‘teacher’, ‘instructor’ or ‘facilitator’, who provides feedback on non-technical skills during medical team training, using a scenario-based ‘immersive simulation’ in a (near) authentic setting.

### Development and testing

The survey was constructed based on available literature regarding faculty development and observation of non-technical skills as well as interviews with five experts: an experienced psychologist in human factors and behavioural skills (WvL), two experienced simulation facilitators (AB, WvM) and two educational specialists (DV, DD). The survey was pilot-tested by seven participants who were excluded from participation in the final survey. Two double questions were split into separate questions, several small textual changes were made and one question was removed due to inconsistent interpretation.

The final survey consisted of five clusters: (1) demographic data, (2) level of experience, (3) observational aspects and difficulty, (4) observation strategy and (5) level of education and training. The survey consisted a total of 42 questions (including the consent questions), with the following distribution: fourteen multiple choice questions, nine open-text questions, twelve Likert-scale questions, three slider-scale questions and four matrix table questions. The exact number of questions presented to the participant was dependent on the provided answers as ‘adaptive questioning’ would only present relevant questions based on previous given input to minimise drop-out.

Translations were done by three different native speakers who all had academic language proficiency (i.e. Dutch-French, Dutch-German and Dutch-English).

### Ethical review board and informed consent

The study was approved by the independent Dutch Association for Medical Education Ethical Review Committee (ID 2019.1.5).

The participant’s information letter was included in the distributive e-mails sent out to the participants. This letter included the aims and goals of the study, participants’ anticipated efforts (anticipated survey completion of 15 min), participants’ rights for withdrawal, data handling, ethical approval, research team information and contact details. At the start of the survey, participants were presented with an option to review this information letter, followed by an active opt-in informed consent question. Participation was anonymous; all demographic data used for descriptive analyses regarding age group, gender, background, country of employment and type of their organisation were voluntary questions.

### Data collection

The survey was distributed as an ‘open survey’ via e-mail through local, regional and national simulation centres and organisations using a non-discriminative snowball sampling method. The survey link was open for responses between May and November 2019. Participants had 1 week to complete their survey. Afterwards, the system would close their record and mark their attempt as ‘incomplete’. Partially completed surveys were included for analysis to limit non-response bias. Raw data were stored in Qualtrics online platform (Qualtrics™, Provo, UT, USA). Respondents were prevented from taking the survey more than once using Qualtrics build-in functionality, which used cookies to prevent users from accessing the survey link more than once.

### Data analysis

Quantitative data were analysed using descriptive statistical analysis using IBM Statistic Package for the Social Sciences (IBM Corp.© SPSS Statistics for Windows, Version 28.0.0.0). A data entry record with no questions answered past the informed consent and demographic section was regarded a dropout. Level of experience was calculated as the amount of days a facilitator had observed non-technical skills. Exploration of the association between level of experience and Likert-scale results were analysed using Spearman’s Rho test. Qualitative data on open-ended questions were analysed by applying open, axial and selective coding by two researchers (GB, LM) independently using an iterative, constant comparison approach. Any discrepancies in coding were discussed until a consensus was reached.

## Results

**Fig. 1:**
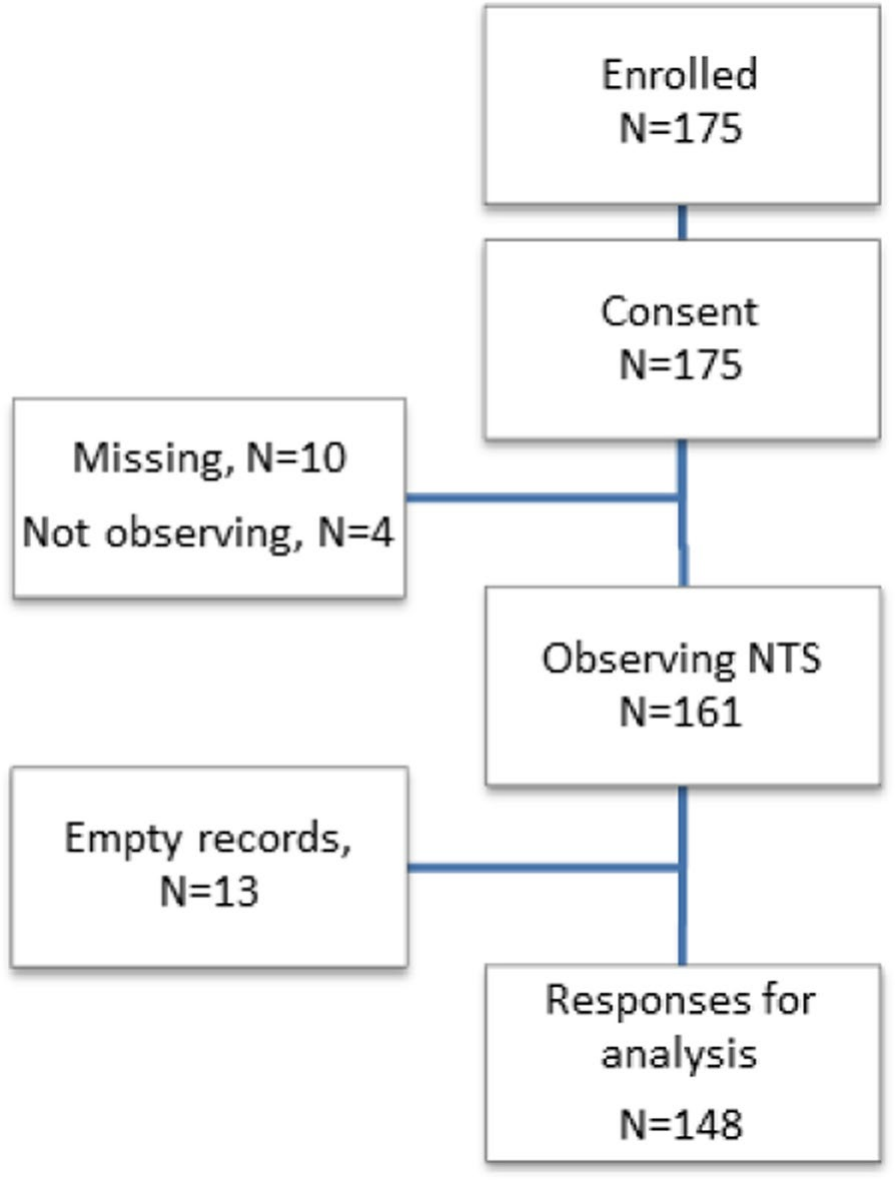
Overview of responses

After a 7-month period, 175 people had participated in the survey. Twenty-seven participants were excluded either because of dropout (*N* = 23, 13%) or non-observation of NTS (*N* = 4, 2%), leaving a total of 148 responses for analysis (Fig. 1).

Table 1 provides an overview of participants’ demographic data. The sample included a variety of facilitators, both men (51.3%) and women (48.0%) aged between 20 and 69 years. Medical doctors (48.6%) and (registered) nurses (41.2%) made up the vast majority of respondents. Participants were employed in over seventeen countries, with the majority working in the Netherlands (51.4%). Most respondents were employed (only) in university medical centres (44.6%), teaching hospitals (15.5%) and teaching facilities (23.6%) such as simulation centres or universities of applied sciences. The participants’ experience, calculated as (median, IQR) number of NTS observation days was 216 days [80–520].

**Table 1:** Characteristics of the participants

	*N*	%
**Gender**		
Male	76	51.3
Female	71	48.0
Not specified	1	0.7
**Age category**		
20–29 years	4	2.7
30–39 years	34	23.0
40–49 years	63	42.6
50–59 years	42	28.4
60–69 years	5	3.4
**Job title**		
Medical doctor	72	48.6
(Registered) nurse	61	41.2
Psychologist	4	2.7
Other medical^a^	5	3.4
Teaching - non-medical	5	3.4
Missing	1	0.7
**Country**		
Austria	7	4.7
Belgium	18	12.2
Denmark	10	6.8
France	11	7.4
Germany	9	6.1
Italy	3	2.0
Netherlands	76	51.4
Portugal	2	1.4
UK	4	2.7
Other^b^	8	5.4
**Organisation**		
University medical centre	66	44.6
Teaching hospital	23	15.5
Teaching facility	35	23.6
Multiple	14	9.5
Other^c^	7	4.7
Not specified	3	2.0

^a^Other medical backgrounds included physician assistant, midwife and perfusionist

^b^Other countries included Albania, Finland, Ireland, Norway, Poland, Spain, Switzerland and Turkey

^c^Other organisations included non-teaching hospitals, ambulance services and defence forces

### Self-reported importance and difficulty of NTS

Table 2 provides the results of the respondents’ perceived importance and difficulty of various NTS items. Perceived importance (scales 1–5; mean [SD]) ranged from very important for ‘coping with stress and fatigue’ (score 3,7 [0.8]) to extremely important for ‘communication’ (score 4,8 [0.4]). Perceived difficulty on the other hand (scale 1–5; mean [SD]) ranged from slightly difficult for ‘leadership’ (score 1,8 [0.8]) to moderately difficult for ‘coping with stress and fatigue’ (score 3,1 [1.1]). Spearman’s rho analysis showed no association between the level of experience and any of the NTS items.

**Table 2:** Perceived importance and difficulty of different NTS items

	**Importance**	**Difficulty**
	(Scale 1–5)^a^	(Scale 1–5)^a^
	**Mean (SD)**	**Mean (SD)**
Communication	4.8 (0.4)	1.9 (1.0)
Teamwork	4.6 (0.6)	2.0 (0.9)
Situation awareness	4.5 (0.7)	2.6 (1.1)
Leadership	4.4 (0.7)	1.8 (0.8)
Decision-making	4.4 (0.7)	2.0 (1.0)
Attitudes	3.9 (0.8)	2.5 (1.2)
Task management	3.8 (0.8)	1.9 (0.8)
Risk management	3.8 (0.8)	2.9 (1.0)
Coping with stress and fatigue	3.7 (0.8)	3.1 (1.1)

^a^Scale: (1) not at all, (2) slightly, (3) moderately, (4) very and (5) extremely important/difficult

Participants were asked to provide an explanation on why certain non-technical skills were perceived—slightly to moderately—difficult to observe. Qualitative analysis of these responses (*N* = 93) revealed three major themes as shown in Table 3.

**Table 3:** Arguments for perceived difficulty in NTS observation

Theme	Quotes
**1. Not everything can be observed** The availability of NTS to be observed is influenced by the -scripted- training scenario and the degree of (non)verbal expression by the participants. E.g. certain NTS cannot be observed directly as they are mental processes of the participants.	Translated: “Observation is what you see, without knowing what the intentions and thoughts of the observed [participants] are” [R6] “Some people are more introvert … and do not think/talk out [loud] in the room. Also some have vague mimics and gestures.” [R126] Translated: “Depending on the scenario (complexity, length, roles), some NTS are not addressed.” [R24] Translated: “Duration of the scenarios is sometimes too short.” [R70]
**2. Not everything is observed** Even when NTS are expressed, not everything is actually seen or heard by the facilitator. Many variables influence facilitators’ opportunity and capability of NTS observation, hence NTS aspects can be missed easily.	Translated: “Especially in larger teams (over 3 persons) it is difficult to capture all NTS. Most of the (non-verbal) communication happens simultaneously between team members. I therefore focus especially on the team leader.” [R4] “Simulation situations are many times very loud and it is difficult to hear all the communication which occurs.” [R124] Translated: “More subtle variants [of NTS] that do influence the interaction between people, can be missed.” [R40] Translated: “It [observation of NTS] is very strenuous, at the end of a training day you sometimes miss things.” [R16]
**3. Interpretation of the observed NTS is difficult** Whenever NTS are actually expressed and observed by the facilitator, interpretation and analysis is difficult and subjective to personal influences	Translated: “Observations are often subjective and influenced by your own frame of reference and experience.” [R9] “They [certain NTS] are more difficult to observe because you cannot have a list of unique behavioural markers. There may be more ways to achieve a good goal.” [R123] Translated: “Many NTS items are interlinked and influence each other, which makes them hard to unravel.” [R43] Translated: “Because [the training] takes place in a simulation center, people may (re)act different compared to the authentic work-place setting.” [R23]

#### Theme 1: Not everything can be observed

Not all aspects of non-technical skills are expressed to the facilitator, and therefore, some cannot be observed directly. As an example, ‘mental processes’ or ‘mental frames’ of the participants are of utmost importance but cannot be observed directly. Also, the scripted training session itself might limit the expression of certain non-technical skills, e.g. due to time constraints or a particular focus of the training (i.e. scenario script).

#### Theme 2: Not everything is observed

Even if non-technical skills are expressed (either verbally or nonverbally), they can be missed easily by the facilitator, e.g. because the facilitator is focusing on a (pre)specified other aspect. It might also occur due to the subtlety of the expression, multiple expressions at the same time, hearing difficulties (e.g. audio difficulties of the simulation setting) and/or fatigue of the facilitator.

#### Theme 3: Interpretation of the observed NTS is difficult

Even when expressions or behaviours are observed by the facilitator, subsequent interpretation and analysis are difficult. Reported reasons for this are as follows: (1) interpretation is a ‘subjective process’, influenced by the facilitators’ own frame of reference and/or experience; (2) not all NTS have clear standards and/or observable criteria, e.g. ‘attitudes’ are reported to be (more) difficult to observe due to this phenomenon; (3) interpretation might be complex as performance fluctuates over time during the training session; (4) non-technical and technical skills might be intertwined hampering cause-effect interpretation and (5) participants might express different behaviour compared to their authentic workplace setting.

When participants were asked to indicate the percentage of observed NTS (in relation to the amount of NTS being expressed) in their average training session on a sliding scale (0–100%), and the mean [SD] result is 57% [17.6]. Furthermore, the mean [SD] number of team members facilitators observed, outnumbered their personal reported maximum 5.5 [2.0] and 3.9 [1.8], respectively; *t*(122) = 8323; *p* < 0.001). This finding was universal amongst all facilitators and not associated with the level of experience within the sample population (*ρ* = 0.084, *n* = 122, *p* = 0.360).

Facilitators were asked to elaborate on the barriers and enablers regarding the observation of NTS. Qualitative analysis (*N* = 106) revealed some overlap with the observational difficulties previously described. In addition, ‘other concomitant tasks’ such as role play in the scenario (i.e. patient, senior staff member) or observation of technical skills were most often cited as important barriers. Quote: [Translated] ‘You have to perform multiple tasks as a facilitator: provide information [to the participants], [scenario] role play, control the technology [i.e. mannikin] and make observations, so you cannot pay full attention to the observation of NTS.’ [R43] Reported enablers on the other hand included the use of multiple (co-)observers, having predetermined learning goals to focus on during the observation and making use of video recordings. Quote: [Translated] ‘There is a lot happening in a very short time span and I also need to focus on the content. I am always glad there are two of us.’ [R21] Furthermore, personal knowledge and experience were mentioned as well for identifying relevant behavioural skills: e.g. ‘It’s very hard to pick up on everything, training [and] practicing help[ed] to improve skills at recognition.’ [R115].

### Observation strategies and tools used for observing NTS

**Fig. 2:**
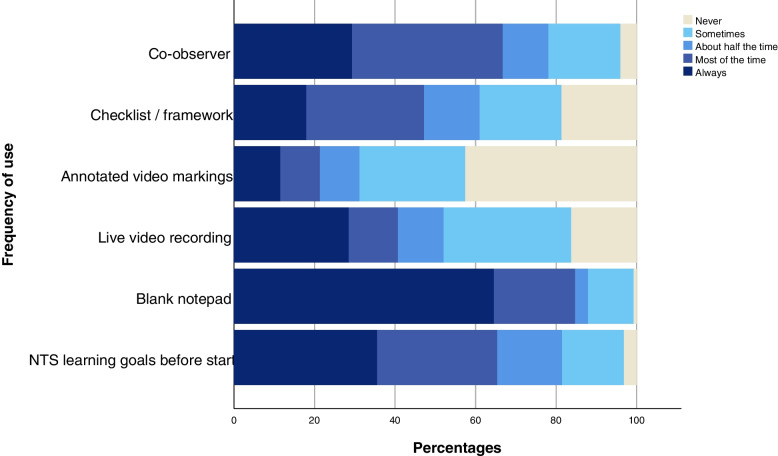
Strategies and tools used in the observation of NTS

Several strategies (e.g. co-observer, predefined learning goals) and tools (e.g. checklists/frameworks) can be utilised while observing NTS. The frequency by which health care facilitators used these is visualised in Fig. 2 (see also Supplementary Table S1).

A blank notepad, co-observers and predefined NTS learning goals were used most frequently. The use of a checklist/framework varied widely: 18% always, 29% most of the time, 14% about half the time, 20% sometimes and 19% never. All participants stating they used a checklist/framework at least sometimes were asked to specify this, including whether they had received formal training in its application (see Supplementary Table S2). The percentage of trained faculty was under 50% (range 0–45%) for each framework or checklist, with the exception of the 15-item CRM framework by Rall et al. [33] (12/14, 86%).

Participants were asked about the perceived usefulness of frameworks for their observation practice. Answers varied from: ‘not at all useful’ 5%, ‘slightly useful’ 23%, ‘moderately useful’ 26% and ‘very useful’ 31% to ‘extremely useful’ 15%. An additional open-ended question was asked to explore this further (see Table 4). Reported benefits of using a checklist/framework were the provision of a structure, a more objective observation and beneficial for research purposes. Reported disadvantages on the other hand were hindering of real-time observation, not suitable for every observation, less useful when more experienced, subjectivity of framework interpretation and resulting in a shift from learner-to-instructor-centred debrief.

**Table 4:** Pros and cons of framework use in NTS observation

In favour of checklist/framework use	In opposition to checklist/framework use
Provides a structure for - Observation - Discussion with co-observers - Debriefing	Hinders real-time observation - Distracts focus - Time-consuming - Does not improve observation
More objective observation - Predetermined behavioural characteristics Useful for research	Not suitable for every observation/training Less useful when more experienced Interpretation remains subjective Tendency to instructor-centred debrief

### Expertise development in NTS observation

Participants were asked to specify their attended training modalities with regard to their expertise development in NTS observation. Observing peers, self-study/own experience and direct supervision by expert(s) were selected most often by 64%, 62% and 45% of participants, respectively. The usefulness score (mean, [SD]) on a 5-point Likert scale was highest for direct supervision by expert(s), 4.21 [0.67], as shown in Table 5.

**Table 5:** Expertise development in NTS observation

	**Training**	**Usefulness**	**Competence**
		Scale (1–5)^a^	Scale (1–5)^a^
	***N* (%)**	**Mean (SD)**	**Mean (SD)**
Self-study/experience	92 (62%)	3.70 (0.98)	
Observing peers	95 (64%)	4.21 (0.67)	
Direct supervision by expert(s)	66 (45%)	4.42 (0.63)	
Checklist/framework training	48 (32%)	3.87 (0.91)	
Specific NTS observation program	52 (35%)	4.12 (0.95)	
Overall competence			3.42 (0.71)

^a^Scale: (1) not at all, (2) slightly, (3) moderately, (4) very, and (5) extremely useful/competent

Participants were given the option to comment on what aspects were most contributing to their learning curve with regard to the observation of NTS. Qualitative analysis (*N* = 53) is in line with the quantitative data as ‘reflection on one’s individual observation, either by peers or experts’ is mentioned by 49% (26/53). Quote: [Translated] ‘Observing the same scenario together and discussing what you have seen [is most useful].’ [R25].

Overall, given their faculty development, participants perceived themselves as ‘moderately competent’ for observing NTS (mean [SD] 3.42 [0.71]). There was no association between the level of experience and self-perceived overall competence (*ρ* = 0.122, *N* = 120, *p* = 0.185).

Finally, participants were asked whether they had future training needs (Question: ‘Are there any aspects you would like (additional) training for regarding the observation of NTS’). A total of 48% (57/119) answered positively whereas 52% (62/119) declined on having future training goals. There was neither an association between future training needs and self-perceived overall competence (r_pb_ − 0.125, *N* = 119, *p* = 0.099), nor with the level of experience (r_pb_ 0.128, *N* = 119, *p* = 0.167). Respondents who answered positively on having future training aspects were asked to specify these in an open-ended question. The majority of these answers related to either receiving direct feedback from other peers/experts, e.g. [Translated] ‘… More direct supervision and observation from trainers’ [R18] and [Translated] ‘I would like to see a training with side-by-side NTS analysis by participants and experts of some pre-recorded video fragments.’ [R25] or related to ‘training benefits’ in general, e.g. [Translated] ‘Extra training is always beneficial’ [R20]. Two other interesting quotes stood out: ‘I would like to have [had] formal training during my early practice as [an] observer.’ [R120] and [Translated] ‘…repetitive NTS observation training with some certification as many people are currently observing NTS without any proof of education/training’. [R36].

## Discussion

This study endorses the opinion that observation of non-technical/behavioural skills is a complex task also within the medical community. The main results of the three explored research questions will be discussed consecutively in the sections below.

Medical facilitators regard all of the behavioural skills at least ‘very important’ to observe. Given the corresponding (only) slight to moderate difficulty with these observations, one can think that observation of behavioural skills is at first sight a simple task. However, when questioned more deeply, three themes were distilled, explaining why observation of behavioural skills is a rather challenging task. First, *not everything can be observed*; mostly mental frames of the participants cannot be observed directly and need to be explored based on the observed subsequent behaviour which is in line with publications recommending facilitators to question participants’ rationale for their actions in the debrief (i.e. ‘double-loop learning’) [18, 24, 34–37]. Second, *not everything is observed*; despite the expression of a behavioural skill, it might not have been observed by the facilitator. This might be intentionally by a distinct focus of the facilitator or unintentionally due to the subtlety or simultaneously of other behavioural skills or from distractions of concomitant tasks. Interestingly, facilitators report that the amount of team members they observe on their average training outnumbers their personal maximum. Salas et al. [27] advocated already that one facilitator should observe only two team members in complex teams so as not to overlook any interactions. With the average amount of team members being almost twice as high, it might not be a surprise that facilitators self-report they capture only half of the expressed behavioural skills [38].

Third, even when behavioural skills are observed by medical facilitators, *interpretation is difficult*, e.g. due to the personal frames of reference from the facilitators. This difficulty is in line with previous publications on limited inter-rater agreement, especially in larger, more complex, multidisciplinary teams [39, 40]. Gingerich et al. [41] however described nicely how to look at this facilitators’ cognition from different research perspectives, including the meaningful idiosyncratic stance, stressing that different interpretations between facilitators are not necessarily a bad thing and combining information from multiple observers and perhaps even participants itself can offer advantages for the debriefing phase [27, 41–44]. The technique of having multiple observers for ‘co-debriefing’ is nicely described by Cheng et al. [45] and appears to be used often by facilitators in our study.

The majority of facilitators had predefined behavioural skills learning goals at the start of their training. This allows for a tailored scenario selection and provides the facilitator with a clear aspect to focus on, also recommended by Salas et al. [27]. Although many checklists/frameworks are available for observation of behavioural skills, its use varied particularly within our population as did their reasoning for (not) using them. We hypothesised that novice facilitators would use some checklist/framework to structure their observation; however, we could not find an association between checklist/framework use and level of experience. We might have missed ‘true novice’ facilitators and/or the perceived disadvantages of checklist/framework use outweigh their perceived benefits in everyday practice. Interestingly, of all the participants that used a checklist/framework, only a minority used well-validated frameworks published in the literature, a phenomenon also addressed by Jenkins [46]. Frameworks can indeed have conflicting properties as reported in our study, i.e. sub-team specific frameworks (e.g. non-technical skills for surgeons [47], scrub practitioners [48] or anaesthetists [49]) have more clearly described behaviours, helping facilitators’ interpretation whereas ‘whole team’ frameworks (e.g. clinical teamwork scale [50], coordination behaviour in acute care teams [51]) are wider applicable possibly benefiting facilitators by gaining experience quicker. Jepsen et al. [8] provided a nice overview of 23 frameworks applicable to the observation of behavioural skills. Either way, facilitators need to provide their learners with detailed feedback to enhance meaningful learning [38] and behavioural frameworks might serve as a ‘cognitive aid’ and prevent facilitators’ fixation. On the other hand, if the observational task is already perceived as too challenging, having to complete yet another (concomitant) task might feel burdensome rather than helpful. Although the impact of checklists/frameworks on feedback and student learning is beyond our scope, using identical frameworks with multiple observers, it could facilitate an inter-rater discussion, thereby enhancing facilitators’ insight into their personal frame of reference.

When it comes to expertise development in behavioural skill observation, the majority of the facilitators were auto-didacts, who acquired their observation skills through either self-study/experience or from observing peers. Given the facilitators’ overall self-perceived moderate competence score coupled with the likely selection bias, it is interesting that only half of the facilitators reported future training desires. A study exploring simulation instructors’ self-reported needs, revealed that a ‘sense of competence’, i.e. achieving their highest level of professional functioning through self-reflection, was indeed present amongst many [52]. Since the vast majority of our sample population was made up of doctors and nurses being actively employed in health care facilities, time constraints and/or workload might be a relevant hypothesis to examine further, especially when striving for (regularly) trained faculty [53, 54]. Deployment of nonclinical faculty has been proposed to overcome time constraints for expertise development and re-certification within the medical domain [53, 55].

### Strengths and limitations

To the authors’ knowledge, this study is the first to address self-perceived current practices and expertise development of behavioural skills observation amongst health care facilitators. The study provides relevant insights for different behavioural (research) domains such as expertise development, implementation of observational frameworks and organisation of (medical) team training. In order to focus on observation and interpretation of behavioural skills, we purposefully did not include questions on team feedback (i.e. ‘debriefing’). One of the important disadvantages of this study methodology is the inability to validate self-reported data. In addition, the cross-sectional sample strategy most likely skewed our sample population towards the more intrinsically motivated facilitators. Quantification of a response rate to analyse this further is unfortunately not possible since there is no formal registration for health care facilitators observing behavioural skills within Europe. In addition, half of the sample population was employed in the Netherlands, although we could not find this to be a confounding variable in the results.

### Future research directions

More insight into what determines qualitative behavioural skill observation would allow tailoring of facilitators’ expertise development and guidelines for everyday practice. For example: in what way does the facilitator-team member ratio influence the quality of the observed behavioural skills? Is the amount of self-reported observations by the facilitators correct, and if so, what behavioural aspects are observed and why? In addition, medical facilitators tend to be mostly auto-didacts who appreciate co-observation with peers or experts. Research analysing how facilitators learn from each other and experts while observing medical teams can further benefit expertise development.

### Future practice directions

Meanwhile, the three themes distilled in this study hold important implications for everyday practice. First, since not everything can be observed directly, facilitators need to reserve sufficient time for the (learner-centred) debriefing to unravel the—unobservable—participants’ thoughts. Second, the shortcomings of the human facilitator should be acknowledged. Facilitators cannot fulfil concomitant tasks in addition to observing behavioural skills and might even limit their attention further to (only) a (pre)specified aspect. This also necessitates multiple faculty members to run a (complex) team training, such as an ‘operator’ for controlling the mannikin and multiple co-observers. Despite this, facilitators need to be aware of their personal frame of reference compared to that of others.

## Conclusions

Observation of behavioural skills by facilitators in health care remains a complex and challenging task. Facilitators’ limitations with respect to attention, focus and (in)ability to do concomitant tasks need to be acknowledged. Although strategies and tools can help to facilitate the observation process, they all have their limitations and are used in different ways.

### Supplementary Information

**Additional file 1: Table S1.** Frequencies of strategies and tools used by facilitators. **Table S2.** Frequencies of different checklists/frameworks used by facilitators.

## Data Availability

The dataset of the current study is available from the corresponding author upon reasonable request.

## Abbreviation

NTS: Non-technical skills

## References

[1] Mazzocco K, Petitti DB, Fong KT, Bonacum D, Brookey J, Graham S (2009). Surgical team behaviors and patient outcomes. Am J Surg.

[2] Siu J, Maran N, Paterson-Brown S (2016). Observation of behavioural markers of non-technical skills in the operating room and their relationship to intra-operative incidents. Surgeon.

[3] Gawande AA, Zinner MJ, Studdert DM, Brennan TA (2003). Analysis of errors reported by surgeons at three teaching hospitals. Surgery.

[4] Kohn LT, Corrigan JM, Donaldson MS, Institute of Medicine Committee on Quality of Health Care in A (2000). To err is human: building a safer health system. To err is human: building a safer health system.

[5] Dedy NJ, Bonrath EM, Zevin B, Grantcharov TP (2013). Teaching nontechnical skills in surgical residency: a systematic review of current approaches and outcomes. Surgery.

[6] Flin R, O'Connor P, Crichton M (2008). Safety at the sharp end. A guide to non-technical skills.

[7] Flin R, Maran N (2015). Basic concepts for crew resource management and non-technical skills. Best Pract Res Clin Anaesthesiol.

[8] Jepsen RMHG, Østergaard D, Dieckmann P. Development of instruments for assessment of individuals’ and teams’ non-technical skills in healthcare: a critical review. Cogn Technol Work. 2015;17(1):63–77.

[9] Murphy P, Nestel D, Gormley GJ (2019). Words matter: towards a new lexicon for 'nontechnical skills’ training. Adv Simul (Lond).

[10] Sevdalis N, Hull L, Birnbach DJ (2012). Improving patient safety in the operating theatre and perioperative care: obstacles, interventions, and priorities for accelerating progress. Br J Anaesth.

[11] Schmitt M, Blue A, Aschenbrener CA, Viggiano TR (2011). Core competencies for interprofessional collaborative practice: reforming health care by transforming health professionals’ education. Acad Med.

[12] Manser T (2009). Teamwork and patient safety in dynamic domains of healthcare: a review of the literature. Acta Anaesthesiol Scand.

[13] Julia Neily J, Mills PD, Young-Xu Y, Carney BT, West P, Berger DH (2010). Association between implementation of a medical team training program and surgical mortality. JAMA.

[14] Catchpole KR, Dale TJ, Hirst DG, Smith JP, Giddings TA (2010). A multicenter trial of aviation-style training for surgical teams. J Patient Saf.

[15] Marshall DA, Manus DA (2007). A team training program using human factors to enhance patient safety. AORN J.

[16] Awad SS, Fagan SP, Bellows C, Albo D, Green-Rashad B, De la Garza M (2005). Bridging the communication gap in the operating room with medical team training. Am J Surg.

[17] Carpenter JE, Bagian JP, Snider RG, Jeray KJ (2017). Medical team training improves team performance: AOA critical issues. J Bone Joint Surg Am.

[18] Kolbe M, Grande B, Spahn DR (2015). Briefing and debriefing during simulation-based training and beyond: content, structure, attitude and setting. Best Pract Res Clin Anaesthesiol.

[19] Issenberg SB, McGaghie WC, Petrusa ER, Lee Gordon D, Scalese RJ (2005). Features and uses of high-fidelity medical simulations that lead to effective learning: a BEME systematic review. Med Teach.

[20] Lederman LC (1992). Debriefing: toward a systematic assessment of theory and practice. Simul Gaming.

[21] Savoldelli GL, Naik VN, Park J, Joo HS, Chow R, Hamstra SJ (2006). Value of debriefing during simulated crisis management: oral versus video-assisted oral feedback. Anesthesiology.

[22] Tannenbaum SI, Cerasoli CP (2013). Do team and individual debriefs enhance performance? A meta-analysis. Hum Factors.

[23] Arora S, Miskovic D, Hull L, Moorthy K, Aggarwal R, Johannsson H (2011). Self vs expert assessment of technical and non-technical skills in high fidelity simulation. Am J Surg.

[24] Burns CL (2015). Using debriefing and feedback in simulation to improve participant performance: an educator’s perspective. Int J Med Educ.

[25] Roussin CJ, Weinstock P (2017). SimZones: an organizational innovation for simulation programs and centers. Acad Med.

[26] Gordon M, Farnan J, Grafton-Clarke C, Ahmed R, Gurbutt D, McLachlan J, et al. Non-technical skills assessments in undergraduate medical education: a focused BEME systematic review: BEME Guide no. 54. Med Teach. 2019:41(7)732-45. 10.1080/0142159X.2018.1562166 30736714

[27] Salas E, Reyes DL, Woods AL. The assessment of team performance: observations and needs. Innovative Assessment of Collaboration. Methodology of Educational Measurement and Assessment; 2017. p. 21–36.

[28] Hull L, Arora S, Stefanidis D, Sevdalis N (2015). Facilitating the implementation of the American College of Surgeons/Association of Program Directors in Surgery phase III skills curriculum: training faculty in the assessment of team skills. Am J Surg..

[29] Baker DP, Dismukes RK (2002). A framework for understanding crew performance assessment issues. Int J Aviat Psychol.

[30] Boet S, Bould MD, Layat Burn C, Reeves S (2014). Twelve tips for a successful interprofessional team-based high-fidelity simulation education session. Med Teach.

[31] Lindqvist SM, Reeves S (2007). Facilitators’ perceptions of delivering interprofessional education: a qualitative study. Med Teach.

[32] Russ S, Hull L, Rout S, Vincent C, Darzi A, Sevdalis N (2012). Observational teamwork assessment for surgery: feasibility of clinical and nonclinical assessor calibration with short-term training. Ann Surg.

[33] Rall M, Gaba D, Howard SK, Dieckmann P. Human performance and patient safety. In: Gropper M, Eriksson L, Fleisher L, Wiener-Kronish J, Cohen N, Leslie K, editors. Miller’s Anesthesia; 2010.

[34] Rudolph JW, Simon R, Raemer DB, Eppich WJ (2008). Debriefing as formative assessment: closing performance gaps in medical education. Acad Emerg Med.

[35] Rudolph JW, Simon R, Rivard P, Dufresne RL, Raemer DB (2007). Debriefing with good judgment: combining rigorous feedback with genuine inquiry. Anesthesiol Clin.

[36] Cheng A, Morse KJ, Rudolph J, Arab AA, Runnacles J, Eppich W (2016). Learner-centered debriefing for health care simulation education: lessons for faculty development. Simul Healthc.

[37] Fey MK, Roussin CJ, Rudolph JW, Morse KJ, Palaganas JC, Szyld D (2022). Teaching, coaching, or debriefing with good judgment: a roadmap for implementing ‘with good judgment’ across the SimZones. Adv Simul (Lond).

[38] Rosen MA, Weaver SJ, Lazzara EH, Salas E, Wu T, Silvestri S (2010). Tools for evaluating team performance in simulation-based training. J Emerg Trauma Shock.

[39] Eppich W, Nannicelli AP, Seivert NP, Sohn MW, Rozenfeld R, Woods DM (2015). A rater training protocol to assess team performance. J Contin Educ Health Prof.

[40] Feldman M, Lazzara EH, Vanderbilt AA, DiazGranados D (2012). Rater training to support high-stakes simulation-based assessments. J Contin Educ Health Prof.

[41] Gingerich A, Kogan J, Yeates P, Govaerts M, Holmboe E (2014). Seeing the ‘black box’ differently: assessor cognition from three research perspectives. Med Educ.

[42] van der Vleuten CP, Schuwirth LW (2005). Assessing professional competence: from methods to programmes. Med Educ.

[43] Govaerts MJ, Schuwirth LW, Van der Vleuten CP, Muijtjens AM (2011). Workplace-based assessment: effects of rater expertise. Adv Health Sci Educ Theory Pract.

[44] Weller J, Shulruf B, Torrie J, Frengley R, Boyd M, Paul A (2013). Validation of a measurement tool for self-assessment of teamwork in intensive care. Br J Anaesth.

[45] Cheng A, Palaganas J, Eppich W, Rudolph J, Robinson T, Grant V (2015). Co-debriefing for simulation-based education: a primer for facilitators. Simul Healthc.

[46] Jenkins B (2015). Training and assessment of non-technical skills in the operating theatre: where next?. Anaesthesia.

[47] Geraghty A, Paterson-Brown S (2020). Non-technical skills for surgeons (NOTSS). Surg Infect (Larchmt).

[48] Flin R, Mitchell L, McLeod B (2014). Non-technical skills of the scrub practitioner: the SPLINTS system. ORNAC J.

[49] Flin R, Patey R (2011). Non-technical skills for anaesthetists: developing and applying ANTS. Best Pract Res Clin Anaesthesiol.

[50] Guise JM, Deering SH, Kanki BG, Osterweil P, Li H, Mori M (2008). Validation of a tool to measure and promote clinical teamwork. Simul Healthc.

[51] Kolbe M, Burtscher MJ, Manser T (2013). Co-ACT–a framework for observing coordination behaviour in acute care teams. BMJ Qual Saf.

[52] Frei-Landau R, Levin O (2023). Simulation-based learning in teacher education: using Maslow’s Hierarchy of needs to conceptualize instructors’ needs. Front Psychol.

[53] Hull L, Arora S, Symons NR, Jalil R, Darzi A, Vincent C (2013). Training faculty in nontechnical skill assessment: national guidelines on program requirements. Ann Surg.

[54] Eva KW, Bordage G, Campbell C, Galbraith R, Ginsburg S, Holmboe E (2016). Towards a program of assessment for health professionals: from training into practice. Adv Health Sci Educ Theory Pract.

[55] Hull L, Sevdalis N (2015). Advances in teaching and assessing nontechnical skills. Surg Clin North Am.

