# Supplementary material for: Observation of behavioural skills by medical simulation facilitators: a cross-sectional analysis of self-reported importance, difficulties, observation strategies and expertise development
Source: Adv Simul (Lond). 2023 Nov 29;8:28. doi: 10.1186/s41077-023-00268-x (PMC10685611; doi:10.1186/s41077-023-00268-x)
Supplement: Supplementary file 1 — Additional file 1: Table S1. Frequencies of strategies and tools used by facilitators. Table S2. Frequencies of different checklists/frameworks used by facilitators. [file 41077_2023_268_MOESM1_ESM.docx]

**Supplementary tables**

**Additional Table S1 (supplement): Frequencies of strategies and tools used by facilitators**

|  | Never | | Sometimes | | Half the time | | Most of the time | | Always | |
| --- | --- | --- | --- | --- | --- | --- | --- | --- | --- | --- |
|  | N | % | N | % | N | % | N | % | N | % |
| Blank notepad | 1 | 1% | 14 | 11% | 4 | 3% | 25 | 20% | 80 | 65% |
| Co-observer | 5 | 4% | 22 | 18% | 14 | 11% | 46 | 37% | 36 | 29% |
| Predefined NTS learning goals | 4 | 3% | 19 | 15% | 20 | 16% | 37 | 30% | 44 | 36% |
| Live video recording | 20 | 16% | 39 | 32% | 14 | 11% | 15 | 12% | 35 | 29% |
| Annotated video markings | 52 | 43% | 32 | 26% | 12 | 10% | 12 | 10% | 14 | 12% |
| Checklist/framework | 23 | 19% | 25 | 20% | 17 | 14% | 36 | 29% | 22 | 18% |

**Additional Table S2 (supplement): Frequencies of different checklists/frameworks used by facilitators**

|  | Never | | Sometimes | | Half the time | | Most of the time | | Always | | % Trained | |
| --- | --- | --- | --- | --- | --- | --- | --- | --- | --- | --- | --- | --- |
|  | N | % | N | % | N | % | N | % | N | % | N | % |
| Anaesthetists Non-Technical Skills (ANTS) | 61 | 62% | 18 | 18% | 3 | 3% | 14 | 14% | 3 | 3% | 0 | 0% |
| Non-Technical Skills for Surgeons (NOTSS) | 90 | 91% | 7 | 7% | 0 | 0% | 2 | 2% | 0 | 0% | 4 | 44% |
| Scrub Practitioners List of Intraoperative Non-Technical Skills (SPLINTS) | 95 | 96% | 1 | 1% | 2 | 2% | 1 | 1% | 0 | 0% | 1 | 25% |
| Oxford Non-Technical Skills (NOTECHS) | 94 | 95% | 2 | 2% | 2 | 2% | 1 | 1% | 0 | 0% | 1 | 20% |
| Team Emergency Assessment Measure (TEAM) | 85 | 86% | 6 | 6% | 4 | 4% | 4 | 4% | 0 | 0% | 3 | 21% |
| Simulation Team Assessment Tool (STAT) | 91 | 92% | 6 | 6% | 0 | 0% | 2 | 2% | 0 | 0% | 2 | 25% |
| Objective Teamwork Assessment System (OTAS) | 94 | 95% | 4 | 4% | 0 | 0% | 0 | 0% | 1 | 1% | 0 | 0% |
| Clinical Teamwork Scale (CTS) | 95 | 96% | 3 | 3% | 1 | 1% | 0 | 0% | 0 | 0% | 0 | 0% |
| 15 CRM points by Gaba & Rall | 85 | 86% | 1 | 1% | 1 | 1% | 1 | 1% | 11 | 11% | 12 | 86% |
| Local framework | 88 | 89% | 3 | 3% | 2 | 2% | 3 | 3% | 3 | 3% | 5 | 45% |
